# Symptoms, Treatment and Pronostic of Encephalic Wounds

**Published:** 1918-12

**Authors:** 


**Symptoms, Treatment and Pronostic of Encephalic Wounds in Fifty-six Observations.** By André MOULONGUET and Pierre LEGRAIN, *Bulletin de la Société de Chirurgie*, June 4, 1918. Presented by A. LAPOINTE.

M. Lapointe considers the study of wounds in the encephalon particularly important. With a great many cases no diffused syndrome or sign of a seat has been detected, but this should not surprise anyone. The author has made his authentication 18 times out of 80 encephalic wound cases. In the treatment, the first important question is "*How far is the primary closing of the scalp good?*" Messrs. Moulonguet and Legrain emphatically declare it dangerous. The wide excision of the lesioned tissues must be the condition of success, though hardly practical here.

Gross and Houdart say that, to ensure the success of a primary closing of the scalp over an encephalic wound, it is sufficient to wash the cerebral seat with warm serum and make a light curettage to bring out clots, bone splinters or projectile fragments. Tanton, who is also a great partisan of primary closing, says complete disinfection of the cranio-cerebralic wounds is one of the most easy to obtain. Willems has maintained the same opinion recently.

Moulonguet and Legrain, however, are not wrong in doubting the sterilizing effect of this surface curetting, for the infectious action of a projectile is never negligible whether in the brain or elsewhere; and, so far, we have no other means of sterilizing an encephalic wound since large excision is not practicable. To primary closing there is another objection. In the wounds of encephalon, which have remained open, the beatings of the brain eliminate secretions, tissues bruised and soiled by the projectile, and the minute splinters which have been missed at the time of surgical intervention. This beating is one of the most active means of defence for the encephalic parenchyma. Primary closing suppresses its curative action.

The great advantage of primary closing would be to preserve the meningo-encephalic seat from all secondary exogenic infections.

The different statistics established after three months of intensive work under Verdun brought the most conflicting conclusions. Gross and Houdart think the lower percentage of fatal cases was due to primary closing; while Moulonguet and Legrain, who did not establish their statistics on selected cases, but on even desperate cases, such as men in the coma, still maintain primary closing is not convincing.

Some technical points deserve attention. Moulonguet and

Legrain find ether or local anesthesia preferable to chloroform. The latter, because of its affinity for the lipoids of the brain, paralyzes the means of defence of the parenchyma against infections, and it is prudent to give it up in cerebral surgery.

The author then proceeds to explain the best methods of extracting small and large fragments from the encephalon. For instance, he says the extraction of projectiles by electro-magnet, apart from being useless in the case of non-magnetic bodies, necessitates a very complicated machinery. (Extracting a projectile from an infested encephalon is a very risky process, and Lapointe declares that he has not had one satisfactory result out of the six cases on which he operated).

Should the cerebral seat be drained? " No ", say Moulonguet and Legrain, for the healthy parenchyma becomes ulcerated at the extremity of the drains.

Lumbar puncture is indicated only in cases of meningitic reaction or hypertension.

From the work presented by Moulonguet and Legrain the teaching is this : primary closing of brain wounds is injurious; primary extraction of deeply lodged projectiles always advisable; and properly thought-out dressings contribute to prevent cerebral hernias. This work is an important contribution to the study of cranio-encephalic wounds.

